# While You Are Sleeping: Marital Ambivalence and Blunted Nocturnal Blood Pressure

**DOI:** 10.3390/ijerph21060723

**Published:** 2024-06-01

**Authors:** Wendy C. Birmingham, Raphael M. Herr, Mikel Cressman, Neha Patel, Man Hung

**Affiliations:** 1 Psychology Department, Brigham Young University, Provo, UT 84602, USA; mikelpc@student.byu.edu; 2 Department of Medical Informatics, Biometry and Epidemiology, Friedrich-Alexander-Universität Erlangen-Nürnberg (FAU), 91054 Erlangen, Germany; raphael.herr@fau.de; 3 College of Dental Medicine, Roseman University of Health Sciences, South Jordan, UT 84095, USAmhung@roseman.edu (M.H.); 4 Department of Orthopedic Surgery Operations, University of Utah, Salt Lake City, UT 84108, USA

**Keywords:** relationship quality, nocturnal dipping, blood pressure, ambivalence

## Abstract

Marital relationships offer health benefits, including a lower risk of cardiovascular disease (CVD). However, quality of the relationship matters; ambivalent behaviors may increase CVD risk by affecting blunted nocturnal blood pressure (BP) dipping. This study tracked daytime and nocturnal SBP and DBP in 180 normotensive individuals (90 couples; participant mean age 25.04; 91.58% white) over a 24 h period using ambulatory blood pressure monitors to explore the impact of martial quality. Results showed that perceptions of spousal ambivalence were associated with blunted nocturnal BP dipping. Perceptions of one’s own behavior as ambivalent also showed blunted nocturnal dipping. When in an ambivalent relationship, a gender interaction was found such that women were most likely to have blunted SBP dipping, but men were more likely to have blunted nocturnal DBP dipping. Overall, this study found an association between ambivalence and BP dipping, thus uncovering one virtually unexplored pathway by which marital relationships may have adverse effects on health.

## 1. Introduction

Marriage confers both physiological and psychological health benefits [1,2], including lower risk of stroke, incidence of cardiovascular disease (CVD), hypertension, CVD mortality, mortality after myocardial infarction, telomere shortening, and early-onset dementia [1,3,4,5,6,7]. But the quality of the marriage matters [8,9,10,11,12]. Marriages that are high quality are associated with fewer illness symptoms, lower mortality risk, lower depression, and lower cardiovascular reactivity during marital conflict [9,10]. Low-quality marriages are associated with low marital satisfaction, negative attitudes and behaviors, hostile interactions [13,14,15], lower immune functioning, and greater morbidity and risk for mortality [16,17]. Low-quality marriages are also associated with heightened blood pressure and heightened heart rate during hostile marital interactions and with worse mental health when such interactions are more frequent [11,14,18,19,20,21,22]. Thus, it is not just marriage per se that is beneficial, but it is marriages that are high quality which are health protective, while lower-quality marriages have a deleterious effect on both physical and psychological health [15,23].

Not all marriages, however, are uniformly high quality or low quality. Prior work has generally conceptualized marital quality as ranging from highly positive to highly negative, yet marriages can contain both high positivity (supportive) and high negativity (aggravation) concurrently (ambivalent). In other words, spouses can be sources of support, compassion, and encouragement but can also be sources of conflict, insensitivity, or criticism. Research has shown ambivalent marital behavior associated with greater stress, lower self-disclosure in marital interactions, higher interleukin-6, fibrinogen, and higher C-reactive protein [24,25]. An important biological pathway in which relationship quality impacts health is through the cardiovascular system, and ambivalent relationships have been clearly associated with worse cardiovascular functioning, including greater cardiovascular reactivity in laboratory studies [26,27,28,29,30] and higher systolic blood pressure (SBP) and diastolic blood pressure (DBP) in married couples across the course of the day [31,32], and has been associated with increased risk of coronary artery calcification [33] compared to those with more purely positive relationships. Supportive relationships in low-income marriages also act as a buffer against negative autonomic responses, whereas ambivalent marital behavior offered no such buffering benefits [34]. See Table 1 for a list of associated medical conditions related to blunted nocturnal dipping.

Much of the work on marital relationship quality and blood pressure (BP) has focused on daytime ambulatory [34,35] or clinical BP readings [36]; yet, research shows nighttime (nocturnal) BP to be a better predictor than daytime BP for cardiovascular and total mortality, coronary heart disease, stroke in healthy adults, and in treated and non-treated hypertensive adults [37,38,39,40,41]. However, BP displays a circadian rhythm in which BP decreases during sleep relative to waking hours (nocturnal dipping) [42]. A normal BP circadian rhythm is characterized by a nocturnal decrease of 10–20% compared to the average awake BP [43,44,45], but this decline can be blunted. Blunted nocturnal dipping is defined as dipping less than 10% overnight (“nondipping”). A normal nocturnal dipping profile is principally due to a decrease in cardiac output, or a stable or slight increase in systemic vascular resistance [46]. A blunted profile can be due to a diminished nocturnal decrease in cardiac output, an increase in systemic vascular resistance, or a combination of both [46]. Blunted dipping has been associated with increased risk for cardiovascular events for both normotensive (approximately 2:1) and hypertensive [47] (approximately 5:1) individuals, independent of overall BP [47,48,49,50]. It has been linked to higher risk of cardiovascular disease, reduced kidney function, high renal risk, poorer renal outcomes, as well as greater kidney disease progression in patients with chronic kidney disease [51,52,53,54,55]. Blunted dipping is also associated with increased risk of all-cause mortality and composite kidney endpoint for people with type 1 diabetes [56], while blunted dipping is associated with diabetic peripheral neuropathy [57]. Further, blunted dipping was associated with incidence of diabetes in a 21-year follow up [58]. It is also associated with increased risk of atherosclerotic process in coronary arteries [59], greater odds of development of coronary artery calcium [60], and hypertension-induced organ damage [61], including left ventricular hypertrophy and reduced arterial compliance [45,62]. Blunted dipping has been associated with arterial stiffness and vascular inflammation [63], higher risk of stroke [64], and with greater cardiovascular morbidity and both cardiovascular and total mortality [49,65,66,67]. In fact, blunted dipping is a better predictor of cardiovascular disease than either daytime or nocturnal averages [68]. Of importance, there is evidence that for every 5% increment in the dipping ratio (i.e., night BP/day BP), there is a 20–30% increase in cardiovascular morbidity and mortality [37,69].

Nocturnal dipping can be assessed with ambulatory blood pressure monitoring (ABPM), which chronicles daily BP fluctuations through a large number of readings, allowing for examination of BP during both waking and sleeping hours [70]. The value of ABPM lies not only in the large number of readings but in the ability for collection of BP readings in real-life conditions rather than using clinical or resting readings for assessment. ABPM allows researchers to rule out the white coat effect (elevations attributable to the clinic or office setting) or masked hypertension [71] and provides clearer prognostic information regarding future cardiovascular outcomes [72,73]. Further, ABPM is valuable in diagnosing borderline hypertension and identifying nocturnal hypertension [74]. Prior ABPM studies have shown wake, sleep, and 24 h ambulatory BP values have greater reproducibility than resting or clinical BP readings [75,76] and provide a more accurate estimate of the patient’s normal BP and cardiovascular prognosis [66,73,77].

**Table 1:** Disease processes linked to blunted nocturnal blood pressure dipping.

Cardiovascular	Kidney Disease	Diabetes
Potential Disease	Potential Disease	Potential Disease
**INCREASED CARDIAC EVENT RISK FOR BOTH NORMOTENSIVE (2:1) AND HYPERTENSIVE INDIVIDUALS (5:1) [48,49,50,51,52].** **GREATER CARDIOVASCULAR MORBIDITY AND TOTAL MORTALITY [50,66,67,68].** **INCREASED RISK OF STROKE [65].** **INCREASED RISK OF ATHEROSCLEROSIS, VASCULAR INFLAMMATION, AND REDUCED ARTERIAL COMPLIANCE [60].** **ARTERIAL STIFFNESS AND VASCULAR INFLAMMATION [64].** **INCREASED ODDS OF DEVELOPMENT OF CORONARY CALCIUM [61].** **INCREASED ODDS OF DEVELOPMENT OF HYPERTENSION-INDUCED ORGAN DAMAGE, INCLUDING LEFT VENTRICULAR HYPERTROPHY [43,61,62,63].**	**REDUCED KIDNEY FUNCTION [54,55].** **HIGH RENAL RISK [52].** **POOR RENAL OUTCOMES [56].** **INCREASED RISK OF COMPOSITE KIDNEY ENDPOINT [57].** **KIDNEY DISEASE PROGRESSION IN PATIENTS WITH CHRONIC KIDNEY DISEASE [52,53].**	**INCREASED RISK OF ALL-CAUSE MORTALITY IN TYPE 1 DIABETICS [57].** **DEVELOPMENT OF KIDNEY DISEASE IN TYPE 1 DIABETICS [59].** **ASSOCIATED WITH PERIPHERAL NEUROPATHY [58].** **INCIDENCE OF DIABETES IN A 21-YEAR FOLLOW UP [59].**

Research has examined dipping among racial/ethnic minorities [78,79], individuals of lower socioeconomic income [80,81], those with health conditions that increase cardiovascular risk such as diabetes and obesity [56,82,83], older or elderly individuals, or hypertensives individuals [84,85,86]. Less work has examined marital status or marital quality on nocturnal dipping, specifically in healthy young adults. In their review, Fortmann and Gallo found preliminary evidence of the protective effects of marriage and suggested ecological momentary assessments (e.g., ambulatory blood pressure assessments) might provide more conclusive evidence to substantiate claims of associations between marriage and nocturnal BP dipping. Because marital quality includes ambivalent relationships and these relationships have been associated with cardiovascular function, a main aim of this study is to examine the impact of ambivalent marital behavior in comparison to purely positive marriages on nocturnal BP dipping using ABPM in order to obtain a more complete picture of cardiovascular functioning. Based on prior literature linking social relationships to blunted dipping [87], and the literature linking ambivalent marital relationships to worse daily blood pressure [31,32], we expect perceptions of spousal ambivalence will be associated with blunted nocturnal BP dipping. A second aim is to examine the impact of one’s own ambivalent behaviors. Marital relationship interactions can be complex, involving interpretations and reactions to a spouse’s behavior [88]. Interpersonal theory posits that an individual’s behavior evokes similar behavior from the partner. In other words, warmth evokes warmth and hostility evokes hostility [89]. We thus expect that those who themselves exhibit ambivalent behavior toward their spouse will show blunted nocturnal dipping. Further, based on the literature which has identified sex as an independent predictor of hypertension, [87,90] we expect sex will act as a moderator.

## 2. Materials and Methods

### 2.1. Participants

Ninety-four couples were recruited through a local university, social media, and the community. All data were collected in the US mountain west in an urban, medium income, educated, English-speaking, mostly white community. Four couples withdrew before study completion, leaving ninety couples. All participants were over 21 years of age, heterosexual, married, and currently living with their spouse. The sample was limited to couples without children in the home in order to maintain a more controlled social context. Because physiological measurements were taken, exclusion criteria included any medical conditions with a cardiovascular component (e.g., hypertension, medications with a cardiovascular component; see Cacioppo et al., 1995 [91]). As hypertension and obesity (BMI over 30 is generally considered obese) are highly correlated, participants were required to have a self-reported BMI no higher than 29.9. Participants were also required to have a smart phone in order to complete a diary reading (see Measures below) at each BP reading. Each was given a personalized access code to the diary website. The mean age of participants was 24.85 years (SD = 4.10, range 21–46), and average length of their marriage was 2.99 years (SD = 2.04; range 1–18). Most were white (91.53%) and college educated (46.89% with a college degree or higher; 51.98% currently pursuing a college degree), with 46.88% reporting an income over USD 30,000. Full demographics can be found in Table 2.

### 2.2. Procedure

Eligible participants completed study questionnaires following informed consent. Couples completed relationship quality measures the day of the ABPM placement. Spouses who attended the study together were separated into different rooms when completing the relationship questionnaires in order to ensure confidentiality. Each was assured their answers were confidential. Spouses were not required to attend the ABPM placement on the same day or time, as each might have differing schedules. Three baseline BPs were taken after a 10 min resting time, each one minute apart, after which participants were fitted with an ambulatory BP monitor and given instructions on use, including how to stop a BP reading if required (e.g., in a meeting, driving, etc.). Participants were shown how to stop readings if they chose to end the study. Monitors took readings of both SBP and DBP randomly twice an hour throughout the day and once per hour overnight. The change to overnight readings (once per hour rather than twice an hour) was based on self-reported bedtime. Participants accessed a personalized diary (see Measures below) from their smart phone in order to complete a diary entry each time a BP reading was taken (diary entries were not completed overnight). Each participant provided their normal bedtime, and monitors were set accordingly. Participants returned the equipment the following morning and received compensation. Participants were paid $75 each ($150 per couple).

### 2.3. Measures

#### 2.3.1. Physiological Measures

Ambulatory blood pressure (ABP) was obtained using the Oscar 2 (Suntech Medical Instruments, Raleigh, NC, USA). The Oscar 2 was designed specifically for ABP assessment and has been validated for both SBP and DBP by international guidelines [92]. The monitor was set to randomly take a reading twice an hour during the day and each hour overnight. The Oscar utilizes codes that may signify problems with the estimation of ABP readings. Based on prior research [93], we deleted readings associated with weak Korotkoff sounds, measurement timeout, and air leaks. Outliers associated with artifactual readings identified using criteria by Marler, Jacobs, Lehoczky, and Shapiro [94] were also discarded. These include (a) SBP < 70 mmHg or >250 mmHg, (b) DBP < 45 mmHg or >150 mmHg, and (c) SBP/DBP < [1.065 + (0.00125 × DBP)] or >3.0.

#### 2.3.2. Psychological and Relationship Measures

Diary. A diary entry was completed by the participant for each BP reading during the daytime. Based on prior work identifying items which can impact BP, the diary collected information on generalized control BP variables including posture, consumption of foods or drinks, or activity at the time of the reading. Diary entries were to be completed within 3–5 min following the BP reading. Verification of completion of a diary entry within the required time was provided by a time/day stamp. Readings which were not completed within the 5 min window were discarded. No diary entries were required for the night ABPM readings.

The Social Relationship Index (SRI; [95]) is a self-report version of the social support interview [96], which was used to assess ambivalence in social relationships. For measures of ambivalence, consistent with prior work [24,97,98], we used a scoring system which categorized behavior as either purely positive or as ambivalent during times of needed support. Participants rated their spouse’s behavior on a six-point scale from “Not at all” to “Extremely” for the questions, “How positive is your spouse?” and “How upsetting is your spouse?” during support seeking. One’s own behavior was measured in the same way. We operationalized these relationships as either supportive or ambivalent. As such, a participant who rated their spouse or themselves with greater than a 1 on positivity and only a 1 on negativity was labeled supportive. Rating of spouse or self that was greater than 1 on positivity and greater or equal to a 2 on negativity was labeled as ambivalent. These cut-off points are consistent with prior work and are based on a broader framework [27,31,99,100]. We used this approach rather than a positivity X negativity interaction with continuous ratings as spouses are not typically rated as aversive (i.e., only negative) or indifferent (i.e., both low positivity and low negativity). Thus, by treating spousal or own ratings as continuous variables, these relationship types would be seriously underrepresented. Other relationships might be appropriate for this type of examination (e.g., family relationships, co-workers, neighbors), but this approach would be inconsistent with the model and analytical approach used in prior studies [31,100].

### 2.4. Statistical Analysis

Researchers have categorized nocturnal BP dipping using night–day ratios as follows: dipping (0.8 to 0.9), extreme dipping (≤0.8), non-dipping (0.9 to 1.0), and reverse dipping (>1.0) [51,65]. According to these classifications, in our sample, 14.9% are extreme dippers, 48.9% are dippers, 25.3% are non-dippers, and 10.9% are reverse dippers. This shows a distribution leaning more towards dippers and non-dippers, with the extremes on both ends being relatively similar. Consequently, we addressed nocturnal BP dipping in a binary manner (dippers vs. non-dippers) based on the BP night/day dipping ratio; dippers had a ratio of ≤0.90, and non-dippers had a ratio of >0.90. This approach involved averaging both daytime and nighttime BP readings, the latter taken from self-reported bedtime to rising time.

Data analysis was performed using SAS version 9.4. We calculated descriptive statistics to assess demographics, baseline SBP and DBP, average daily SBP and DBP, and sleeping SBP and DBP. To explore the relationship between relationship quality and nocturnal blood pressure dipping, we employed mixed model regressions (PROC MIXED). Initially, we identified significant predictors of the dependent variable among covariates (age, BMI, posture, consumption of food or drink, and activity since the previous reading) using forward selection methods. Subsequently, we executed regression models incorporating these significant covariates. Finally, mediation analysis was conducted to explore gender differences within the model.

## 3. Results

### 3.1. Preliminary Analysis

We first examined the frequency distribution of ambivalent spouses versus supportive spouses. In total, 74.67% of spouses perceived their spouse’s behavior as ambivalent. In terms of one’s own behavior, 79% viewed their own behavior toward their spouse as ambivalent. These values are consistent with prior work on ambivalence in marriage [25,32,98]. A series of descriptive statistics was run, and sample characteristics are presented in Table 2. Mean SBP for the overall sample was 132.12 (SD = 24.68), and mean DBP was 74.37 (SD = 15.92). Mean daytime SBP was 135.45 (SD = 18.84), with daytime DBP 77.0 (SD = 9.94). Sleep mean SBP was 119.67 (SD = 20.82), and sleep mean DBP was 60.47 (SD = 10.26). The mean number of readings per participant was 37.04 (range 22–46) for the 24 h period. All outliers due to artifactual readings were discarded as noted above. An average of 1.5% of readings per participant were discarded (range 0–7%).

We next examined the impact of demographics on ambivalent marital quality. Results can be found in Table 3.

### 3.2. Main Analysis

As our main aim was to examine the effect of ambivalence on nocturnal BP dipping, we first examined the effect of relationship quality (ambivalent or supportive) on nocturnal BP dipping. Consistent with our expectations, we found perceptions of spousal ambivalence associated with both SBP (*B* = 0.14, SE = 0.03, t(7455) = 5.43, *p* < 0.001) and DBP (*B* = 0.63, SE = 0.04, t(7355) = 15.34, *p* < 0.001) blunting. We also found one’s own ambivalent behavior associated with blunted SBP (*B* = 0.27, SE = 0.03, t(3997) = 7.69, *p* < 0.001) and DBP (*B* = 0.63, SE = 0.06, t(3997) = 10.15, *p* < 0.001) dipping.

An examination of the effect of sex on dipping found sex associated with dipping for SBP (*B* = −0.68, SE = 0.05, t(7540) = −12.91. *p* < 0.001) but not for DBP dipping (*B* = 0.14, SE = 0.08, t(7540) = 1.8, *p* = 0.07). We then examined whether sex acted as a moderator for the association between relationship quality and nocturnal dipping. We found a significant interaction such that women who viewed their spouse’s behavior as ambivalent were more likely to have blunted nocturnal dipping for SBP (*B* = 0.65, SE = 0.05, t(7354) = 12.43. *p* < 0.001) while men who viewed their spouse’s behavior as ambivalent were more likely to have blunted DBP (*B* = −0.34, SE = 0.08, t(7354) = −4.05, *p* < 0.001). Finally, we examined whether one’s own behavior moderated the effect of spousal behavior to nocturnal dipping, but we found no association for either SBP or DBP dipping. See Table 4.

### 3.3. Ancillary Analysis

While we initially approached nocturnal dipping in a binary manner, exploring the extreme dippers and reverse dippers offers valuable insights. By comparing these groups to regular dippers, we investigated their association with relationship quality. Our findings revealed no significant link between relationship quality and SBP for extreme dippers (*B* = −0.01, SE = 0.04, t(5355) = −0.32, *p* > 0.05) or reverse dippers (*B* = −0.11, SE = 0.06, t(5355) = −1.91, *p* > 0.05). However, a significant association was observed between relationship quality and DBP for both extreme dippers (*B* = 0.19, SE = 0.03, t(5355) = 5.37, *p* < 0.05) and reverse dippers (*B* = −0.422, SE = 0.09, t(5355) = −4.43, *p* < 0.05).

## 4. Discussion

The main aim of this study was to assess the impact of ambivalent marital behavior on nocturnal BP dipping in married heterosexual couples using ABPM. Prior work has examined nocturnal BP dipping in various demographics, social situations, disease states [56,80,101,102], and often in older/elderly or hypertensive individuals [51,84,103,104]. Less work has examined nocturnal dipping and marital relationship quality, specifically regarding ambivalent behavior in marital relationships. In the current study, consistent with our hypothesis, perceptions of spousal ambivalent behavior were associated with blunted nocturnal dipping for both SBP and DBP. These finding are also consistent with existing marital quality literature demonstrating that while marriage itself is beneficial for health outcomes [1,2], the quality of the marriage is important [9,105]. Our findings also are consistent with the literature showing the association with marital quality and cardiovascular disease processes [24,31,33,97]. Further, our study extends the marital health literature by demonstrating that the negative health effects of ambivalent marriages can be seen in nocturnal BP dipping, a clear predictor of cardiovascular disease risk.

Although age was not part of our eligibility requirements, our participants were fairly young adults (mean age 24.51 years). All were normotensive, with no cardiovascular conditions such as hypertension, and none were on medications with a cardiovascular component. The majority reported their spouse’s behavior as ambivalent. Much of the prior work on ambivalent relationships and BP has focused on hypertensive individuals, many of whom are middle-aged or older. However, research has shown an increased BP trajectory over time associated with poor cardiovascular outcomes, even 25 years later [106]. This can be important for younger individuals whose trajectory may start much earlier than generally experienced in a more supportive relationship and before they may even consider monitoring their BP.

We also looked at one’s own behavior toward the spouse based on the interpersonal circumplex [89,107]. It is not surprising that we found one’s own behavior to impact nocturnal dipping. The interpersonal circumplex suggests that positive and negative behaviors (warmth and hostility) by one partner invite similar responses. These processes involve interpretation and reactions to interpersonal interactions, and these interpretations are reinforced over time [88]. As partners interact multiple times over the course of their relationship, their behavioral patterns are likely influenced by the behaviors of their spouse. That can create greater complementarity in behavior [107]. We thus looked at one’s own ambivalent behavior and nocturnal dipping and, in line with the interpersonal circumplex, we found that not only are perceptions of one’s spouse’s ambivalent behavior associated with blunted dipping, but one’s own ambivalent behavior is associated with both SBP and DBP blunted nocturnal dipping. In other words, it is not just dealing with a spouse whose behavior is not always positive that impacts your dipping, but knowing you are acting in an ambivalent fashion to your spouse can also impact your dipping.

Our results showed women to have blunted SBP dipping when they report ambivalent behavior from their spouse. Women have been shown to be more sensitive to conflict or stress in marriage, and this negatively impacts their cardiovascular health [8]. Ambivalence contains high negativity along with high positivity, and our results show that this negativity may be every bit as harmful as that seen in conflictual marriages despite the positivity in the relationship.

Further, women have lower SBP than men during early adulthood and slightly lower DBP than men regardless of age [105]. Hypertension is seen less often in women in young adulthood than men until after age 50 when hypertension increases more rapidly. This difference in BP in younger women may contribute to the lower incidence of CVD in women [105]. Of concern then, the women in our sample were mostly in young adulthood but were already showing signs of increased CVD risk through blunted dipping associated with their marriage. As noted above, BP trajectory over time is associated with poor cardiovascular outcomes. Seeing this blunted dipping early can lead to even greater risk as these women age.

It is interesting that men, in contrast to women, showed blunted DBP within ambivalent marriages. This can be problematic for men, as, despite SBP often shown as the stronger blood pressure measure associated with cardiovascular events and mortality [106], cumulative DBP can increase cardiovascular disease risk [107]. Men have shown greater ambulatory mean sleep DBP than women even in normotensive individuals [105], and while DBP nocturnal dipping is not the same as sleep DBP, it is used to calculate nocturnal dipping. It is thus encouraging to know that men’s high-quality marriages can contribute to better cardiovascular health.

The exploration of nocturnal dipping patterns, moving beyond dichotomy to include extreme and reverse dippers, sheds light on the interplay between BP behavior during sleep and the quality of relationships. The approach to categorizing individuals based on their nocturnal BP changes—distinguishing between those extreme dippers and those reverse dippers—offers a more detailed understanding of cardiovascular health dynamics. In terms of SBP, this study reveals no observable link to relationship quality for both extreme and reverse dippers. This indicates that the fluctuations in SBP, irrespective of whether they exhibit a pronounced dip or a rise during sleep, do not have a straightforward relationship with how individuals perceive the quality of their relationship. Conversely, DBP presents a different picture, showing a significant association with relationship quality in both groups. For extreme dippers, a positive association suggests that improvements in relationship quality may be associated with more pronounced decreases in DBP at night. On the flip side, for reverse dippers, a negative association implies that deteriorations in relationship quality could coincide with increased DBP. This differential impact of relationship quality on SBP and DBP in nocturnal dipping patterns highlights the intricate ways in which social and emotional factors may influence heart health, particularly through nocturnal BP regulation. These findings call for a deeper investigation into the physiological mechanisms that mediate these effects, potentially focusing on the roles of stress and emotional support. Such research could unveil insights into how the quality of relationships affects cardiovascular health, particularly through the lens of DBP changes during sleep.

Despite the strength of our study, there are limitations to address. Our sample was predominately white and educated. Our sample was young, healthy, and from one urban region, and the same associations may not be seen in older individuals, in people of color, or in regions other than urban. We also did not assess sleep or sleep-related disorders such as insomnia, sleep apnea, restless leg syndrome, or periodic limb movement, which could have resulted in a poor night’s sleep. Additionally, our sample was taken from a population of individuals who tend to marry young and is thus not necessarily generalizable to couples who live in areas where individuals marry later in life or to those who do marry later in life or those who have been married longer. Nevertheless, despite these limitations, our study adds to the literature of marriage quality and cardiovascular health. Earlier work on the detrimental impact of blunted dipping has been examined in racial/ethnic minorities, those in differing socioeconomic situations, in older individuals, and in those with health conditions such as diabetes or obesity. Our study specifically examined healthy younger married individuals in order to determine the negative impact that ambivalent behavior can have on nocturnal dipping in this specific population. Future studies should examine dipping and relationship quality in couples who have been married for longer periods of time. Future studies should also examine if ambivalence plays a role in dipping in LGBTQ+ relationships, older couples, and in those in more specific diverse sociocultural relationships.

## 5. Conclusions

Blunted nocturnal dipping was associated with those whose spouse exhibited ambivalent behavior. Blunted nocturnal dipping was also associated with those who self-reported they themselves behaved in an ambivalent manner toward their spouse. As blunted nocturnal dipping is an indicator of future cardiovascular disease, efforts to improve relationship behavior for both spouses would be warranted.

## Figures and Tables

**Table 2:** Sample demographic characteristics.

Variable	Mean (Std Dev)		Min	Max	*N*	%
Age (Year)	25.05	4.84	21.00	59.00	180	
Ethnicity						
White					162	91.58
Hispanic/Latino					10	5.62
Asian					2	1.12
Native American					1	0.56
Pacific Islander					1	0.56
Other					1	0.56
Income						
Less than $15,000					34	19.10
15,000–$29,999					60	33.70
$30,000–$49,000					48	26.4
$50,000–$69,000					18	10.11
$70,000 or more					18	10.13
I would rather not report this					1	0.56
Education						
Graduated from high school					2	1.12
Partial college					94	51.67
Graduated from college					56	32.59
Partial graduate-professional school					16	8.99
Graduated from graduate-professional school					10	5.63

**Table 3:** Effect of demographics on relationship quality.

Variable	*B*	SE	*p*-Value
Ethnicity	0.35	0.04	<0.001
Income	−0.01	0.02	0.70
Sex	0.4133	0.05	<0.001
Education	−0.286	0.268	0.286

**Table 4:** Effect of ambivalence on blood pressure.

Variable	*B*	SE	*p*-Value
Spousal ambivalence			
SBP blunting	0.14	0.04	<0.001
DBP blunting	0.63	0.04	<0.001
Own ambivalent behavior			
SBP blunting	0.27	0.03	<0.001
DBP dipping	0.63	0.06	<0.001
Sex			
SBP dipping	−0.68	0.05	<0.001
DPB dipping	0.14	0.08	0.07
Women’s spouse ambivalent behavior			
SBP dipping	0.65	0.05	<0.001
Men’s spouse ambivalent behavior			
DBP blunting	−0.34	0.08	<0.001

## Data Availability

Data are available from authors upon request and IRB approval.
